# Optimized Convolutional Fusion for Multimodal Neuroimaging in Alzheimer’s Disease Diagnosis: Enhancing Data Integration and Feature Extraction

**DOI:** 10.3390/jpm13101496

**Published:** 2023-10-14

**Authors:** Modupe Odusami, Rytis Maskeliūnas, Robertas Damaševičius

**Affiliations:** 1 Department of Multimedia Engineering, Kaunas University of Technology, 51423 Kaunas, Lithuania; 2 Department of Applied Informatics, Vytautas Magnus University, 53361 Kaunas, Lithuania

**Keywords:** Alzheimer’s disease, data integration, feature extraction, multimodal neuroimaging, optimized convolution

## Abstract

Multimodal neuroimaging has gained traction in Alzheimer’s Disease (AD) diagnosis by integrating information from multiple imaging modalities to enhance classification accuracy. However, effectively handling heterogeneous data sources and overcoming the challenges posed by multiscale transform methods remains a significant hurdle. This article proposes a novel approach to address these challenges. To harness the power of diverse neuroimaging data, we employ a strategy that leverages optimized convolution techniques. These optimizations include varying kernel sizes and the incorporation of instance normalization, both of which play crucial roles in feature extraction from magnetic resonance imaging (MRI) and positron emission tomography (PET) images. Specifically, varying kernel sizes allow us to adapt the receptive field to different image characteristics, enhancing the model’s ability to capture relevant information. Furthermore, we employ transposed convolution, which increases spatial resolution of feature maps, and it is optimized with varying kernel sizes and instance normalization. This heightened resolution facilitates the alignment and integration of data from disparate MRI and PET data. The use of larger kernels and strides in transposed convolution expands the receptive field, enabling the model to capture essential cross-modal relationships. Instance normalization, applied to each modality during the fusion process, mitigates potential biases stemming from differences in intensity, contrast, or scale between modalities. This enhancement contributes to improved model performance by reducing complexity and ensuring robust fusion. The performance of the proposed fusion method is assessed on three distinct neuroimaging datasets, which include: Alzheimer’s Disease Neuroimaging Initiative (ADNI), consisting of 50 participants each at various stages of AD for both MRI and PET (Cognitive Normal, AD, and Early Mild Cognitive); Open Access Series of Imaging Studies (OASIS), consisting of 50 participants each at various stages of AD for both MRI and PET (Cognitive Normal, Mild Dementia, Very Mild Dementia); and whole-brain atlas neuroimaging (AANLIB) (consisting of 50 participants each at various stages of AD for both MRI and PET (Cognitive Normal, AD). To evaluate the quality of the fused images generated via our method, we employ a comprehensive set of evaluation metrics, including Structural Similarity Index Measurement (SSIM), which assesses the structural similarity between two images; Peak Signal-to-Noise Ratio (PSNR), which measures how closely the generated image resembles the ground truth; Entropy (E), which assesses the amount of information preserved or lost during fusion; the Feature Similarity Indexing Method (FSIM), which assesses the structural and feature similarities between two images; and Edge-Based Similarity (EBS), which measures the similarity of edges between the fused and ground truth images. The obtained fused image is further evaluated using a Mobile Vision Transformer. In the classification of AD vs. Cognitive Normal, the model achieved an accuracy of 99.00%, specificity of 99.00%, and sensitivity of 98.44% on the AANLIB dataset.

## 1. Introduction

Alzheimer’s Disease (AD) is a neurodegenerative disorder characterized by progressive cognitive decline and memory impairment. Early and accurate diagnosis of AD is crucial for effective intervention and treatment planning. Patients with AD prolong to dementia and lose physiological functions, eventually leading to death. An estimated 55 million people worldwide have dementia, and more than 60% of them live in low- and middle-income countries [1]. It is anticipated that this will increase to 78 million by 2030 and rise to 139 million by 2050 [1]. Metabolic changes in the brain and significant atrophy contribute to the neurodegenerative processes observed in AD. Magnetic Resonance Imaging (MRI) and Positron Emission Tomography (PET) have become very useful for studying the structural and functional changes linked to Alzheimer’s Disease in the neuroimaging field [2].

The pathogenic nature of Alzheimer’s disease manifests in the brain as structural alterations, including anatomical location, cortical thickness, volumetry, and other morphological features [3]. The capacity to quantify these morphological traits using MRI has resulted in an explosion of methodological research in predicting and categorizing AD. MR images show exceptional anatomical information, and hippocampus shrinkage evaluated on a high-resolution T1-weight MRI is an important criterion for the clinical diagnosis of Alzheimer’s disease [4]. For example, volumetric features of sMRI data were utilized for the classification of Early Mild Cognitive Impairment (EMCI) vs. Normal Cognitive (NC) [5]. sMRI cortical thickness and its underlying geometric information were employed for the early detection of AD [6]. The most useful spatial features of GM were extracted from sMRI and further segmented into ninety regions for Late Mild Cognitive Impairment (LMCI) vs. EMCI classification [7]. Extracted gray matter (GM) images from sMRI using CNN architecture were used for the diagnosis and classification of the CN, EMCI, and LMCI groups [8]. While structural imaging captures downstream pathological changes, it is not appropriate for reflecting changes that precede protein deposition [9]. PET imaging with 18F-fluorodeoxyglucose (FDGPET) imaging can capture brain metabolism characteristics to aid in the detection of lesions for AD classification [10]. For example, FDGPET data was used for the automated classification of AD groups [11]. The risk of AD was predicted based on the deep learning model by extracting FDG PET image features [12]. The fusion of various imaging modalities in multimodal neuroimaging holds the promise of offering comprehensive insights into the metabolic and structural changes occurring in AD [9,13,14].

In recent years, there has been a growing interest in leveraging multimodal neuroimaging data to enhance the accuracy of AD classification. Combining information from multiple imaging modalities can provide a more comprehensive understanding of AD [7,15,16,17] in capturing complementary aspects of brain alterations that may not be evident in a single modality. However, effectively integrating these heterogeneous data sources presents a considerable challenge.

A rising number of studies have used MRI and PET data to discover multilevel and multimodal properties by translating regional brain images into higher-level, more compact characteristics. For example, to classify AD, a new composite image was generated by blending the Gray Matter (GM) tissue region of the brain in both MRI and FDG-PET images using mask and registration coding techniques [18]. Likewise, researchers have classified individuals with AD according to their GM density and glucose utilization from MRI and PET, allowing for a more comprehensive and accurate diagnosis of AD [19]. Furthermore, a novel multimodal image-fusion technique designed to merge PET and MRI data was introduced, in which the extracted features are subsequently input into an ensemble classifier [17]. While the automatic pipeline method described in their study utilized techniques such as Free Surfer and affine registration for pixel-level fusion, achieving precise alignment and ensuring that the combined information accurately reflects the underlying neurobiological changes was a challenge. Furthermore, the extraction of relevant features from these fused modalities introduced complexities in terms of feature selection and interpretability. Although the study employed a range of techniques, including ANOVA, scalar methods, and machine learning classifiers, to identify prominent features, the process of discerning which specific features contribute most significantly to the accurate classification of AD stages remained challenging. The three-channel phase feature learning model demonstrated promise in integrating and learning latent representations from multimodal neuroimaging data, even in the presence of data heterogeneity [10]. However, the successful partial resolution of the heterogeneity issue highlighted the complexity of reconciling distinct characteristics of PET and MRI data within the unified framework.

The multimodality latent space-inducing ensemble Support Vector Machine classifier demonstrated the potential for improved AD classification accuracy. The study’s exploration of an ensemble SVM classifier that induced a latent space via multimodality MRI and PET inputs gave a promising avenue for enhancing AD classification accuracy. However, the intricacy lay in the requirement of effectively reconciling and encapsulating the inherent interrelationships present in MRI and PET modalities within this latent space [20]. An image fusion method to combine MRI and PET images into a composite Gray Matter–PET modality for AD diagnosis has been proposed [21]. While the image fusion method demonstrated superior overall performance compared to unimodal methods, it was noted that its performance in terms of sensitivity and specificity sometimes fell short of optimal levels. The fusion process might introduce subtle distortions or uncertainties that affect diagnostic accuracy.

Additionally, researchers have explored the use of multiscale transform approaches to integrate information from multiple modalities consisting of MRI and PET imaging data in the field of multimodal neuroimaging for the diagnosis of AD. Wavelet-based fusion was used to bring together information from the MRI and PET scans to improve spatial resolution and yielded an image with metabolic and anatomical detail coupled with the finest resolution. Despite the coregistration and alignment process, variability in image resolution and information content between MRI and PET was still a challenge [22]. Ensuring accurate registration and fusion requires overcoming differences in image resolution, contrast, and acquisition protocols [23]. The computational complexity of processing and analyzing data across multiple dimensions is a further disadvantage of multiscale transform methods. These methods frequently involve intricate mathematical algorithms and intensive computational operations, making them time-consuming and resource-intensive [24]. In contrast to multilevel feature learning and multiscale transform approaches for multimodal neuroimaging fusion, our proposed method leverages pre-trained convolution neural networks. Consequently, it requires neither a specific dataset for training nor a specialized network. The proposed network is fed with source images, and optimized feature maps are extracted at layer 1 [25]; Maximum Fusion (MF) is used to fuse the extracted feature. Vision transformers have garnered significant attention in the field of computer vision due to their remarkable performance in image classification tasks, demonstrating their ability to capture long-range dependencies within images [26]. Simple ViT models trained faster and better than the original [27]; the performance of ViTs saturated faster when scaled to be deeper and improved image classification accuracy [28], and Mobile ViT allowed for light-weight global processing of information with transformers [29]. Leveraging the success of vision transformers, we employ fused images as inputs to train a vision transformer model for AD classification.

The research paper’s primary contributions are as follows: (1) An MF strategy is designed to fuse the same depth of feature maps of MRI and PET. (2) An optimized convolution technique including variations in kernel size to increase receptive field and the addition of instance normalization, which ensured no bias towards the modality, is designed to improve alignment and integration of MRI and PET data. (3) A novel fusion model of MRI and PET images using MS strategy and optimized convolution network is proposed, which overcomes the shortcomings of multiscale transform fusion methods and enhances the receptive field of the network.

## 2. Materials and Methods

**Figure 1:**
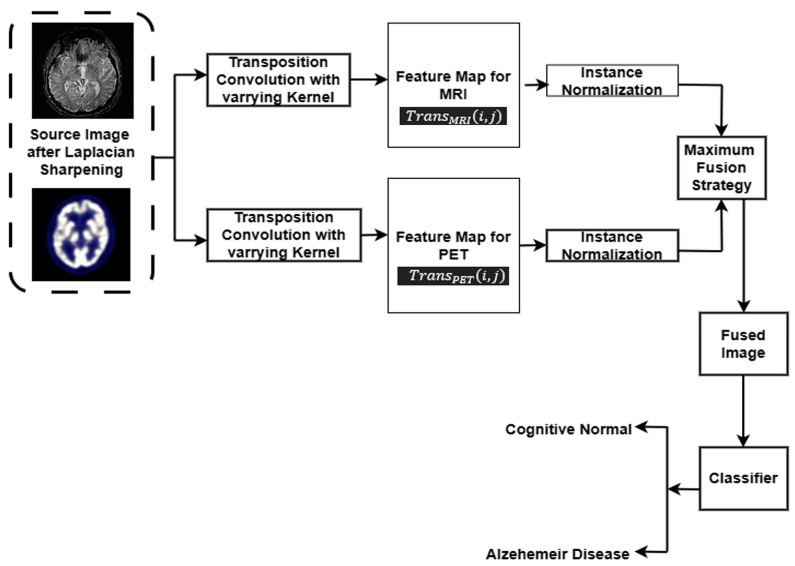
Framework of Proposed Fusion Method.

The Laplacian transform, a mathematical technique for accentuating intricate image details and extracting edge and texture characteristics [30,31], emerges as a pivotal tool in the fusion of MRI and PET images. In this study, MRI and FDG-PET images are collected from three databases (ADNI, OASIS, and AANLIB), with the Laplacian transform demonstrating its significance in efficiently revealing finer aspects of the images, thereby contributing to the improved fusion of MRI and PET modalities. This technique’s potential is well-founded in its ability to capture and highlight subtle features within medical images, aligning with the demands of contemporary image fusion methodologies [32]. This paper uses Laplace sharpening to obtain the fine details of the image. Transposition convolution was optimized by varying the kernel size, and instance normalization is used to extract relevant feature maps from MRI and PET images. The feature maps obtained via MRI and PET are fused using MF to emphasize the strongest activations between the MRI and PET modalities. The proposed fusion model is shown in Figure 1.

### 2.1. Datasets

**Figure 2:**
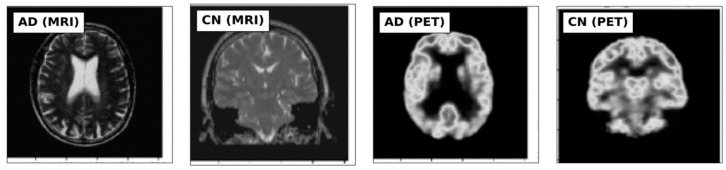
Sample Images from the AANLIB database.

**Figure 3:**
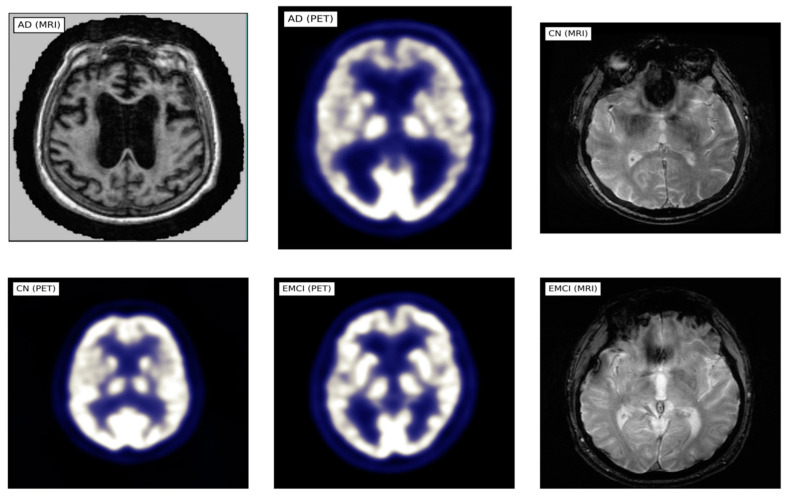
Sample Images from the ADNI database.

**Figure 4:**
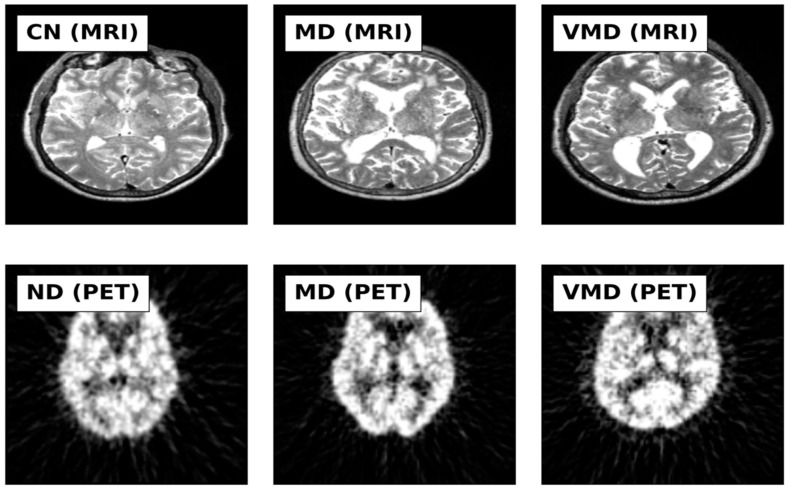
Sample Images from OASIS Database.

This study utilized MRI and PET images obtained from the official website of Harvard University (http://www.med.harvard.edu/AANLIB/home.html (accessed on 15 September 2023)), the ADNI website (https://adni.loni.usc.edu (accessed on 15 September 2023)), and the OASIS website. The brain images under consideration are categorized into two distinct stages, namely Cognitive Normal (CN) and Alzheimer’s Disease (AD). 50 images of each of the stages are downloaded from each website, making a total of 300 images. PET images are in red, green, and blue (RGB) while MRI images are in black and white. Figure 2, Figure 3 and Figure 4 show sample the datasets used from AANLIB, ADNI, and OASIS database, respectively.

### 2.2. Laplace Sharpening

Fine details are obtained from the source image Sn (n=1, which means MRI image; n = 2, which means PET image). The Sn with pixel intensities is represented as $Sn\left(x,y,c\right),$ where (x,y) are the coordinates and c is the color channel [30]. A Gaussian blur kernel is applied to the source image Sn(x,y,c). This is represented by Equation (1):

(1) $$K_{gaussian}=\frac{1}{16}\left(\begin{array}{ccc} 1 & 2 & 1\\ 2 & 4 & 2\\ 1 & 2 & 1 \end{array}\right),$$

The blurred image is then obtained by convolving the source image with the Gaussian blur kernel. This process is expressed in Equation (2): The obtained image (blurred image) after Equation (1) is applied to Si, as is represented in Equation (2).

(2) $${Blurred}_{image}\left(x,y,c\right)=\sum_{i=-1}^{1}\sum_{j=-1}^{1}S_{n}\left(x+i,y+j,c\right).\;K_{gaussian},$$

where: i and j iterate over the 3 × 3 neighborhood of the pixel at position (x,y,c).

To obtain the finer details, a Laplacian kernel, given by Equation (3), is utilized.

(3) $$K_{laplacian}=\frac{1}{16}\left(\begin{array}{ccc} -1 & -1 & -1\\ -1 & 8 & -1\\ -1 & -1 & 1 \end{array}\right)a,$$

By convolving the Laplacian kernel from Equation (3) with the previously obtained blurred image, we generate the Laplacian image. This process is outlined in Equation (4):

(4) $${Laplacian}_{image}\left(x,y,c\right)=\sum_{i=-1}^{1}\sum_{j=-1}^{1}B_{image}\left(x+i,y+j,c\right).\;K_{laplacian}\left(i,j\right),$$

The sharpened image is derived by combining the source image *Sn* (*x*,*y*,*c*) with the Laplacian-filtered image. This combination is controlled by an enhancement factor ‘*k*’. The resulting equation is given by:

(5) $${Sharpened}_{image}\left(x,y,c\right)=Sn\;(x,y,c)+k.\;{Laplacian}_{image}\left(x,y,c\right),$$

Lastly, to ensure that pixel values are within the valid range of [0, 1], the sharpened image is clipped using the following equation:

(6) $${Sharpened}_{image}\left(x,y,c\right)=\mathrm{clip}{(Sharpened}_{image}\left(x,y,c\right),0,1),$$

**Figure 5:**
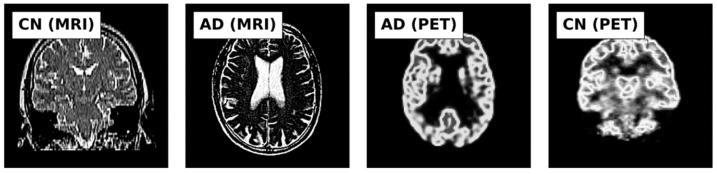
Sample Images after Laplace Sharpening.

Sampled sharpened images from the AANLIB database are shown in Figure 5.

### 2.3. Basic Image Feature Map Extraction Based on Optimized Transposition Convolution

The proposed method leverages the VGG19 network as a backbone for feature extraction due to its exceptional performance in various computer vision tasks. Previous research in the field of image fusion has reported successful results using VGG19 for image fusion tasks. For instance, infrared and visible images were integrated using VGG19 to extract relevant features, and the MF rule was utilized for the final fused image [33,34]. Likewise, transposed convolution was used for upsampling in image resolution to enhance effective feature extraction [35]. In our proposed study, the initial step involves applying transposed convolution after the first convolution layer [36] by varying kernel sizes. Traditional transposed convolution uses fixed-size kernels for upsampling, which might not capture all levels of detail effectively. By varying the kernel size, the convolution operation can adapt to different spatial scales present in the input image [37,38]. This adaptability is particularly useful for retaining fine-grained information while ensuring that larger structures are also captured [39]. Equation (7) gives the transposition operation on the input images.

(7) $$Trans\left(i,j\right)=\sum_{m=-k}^{k}\sum_{n=-k}^{k}Kernel\left(m,n\right).\;X_{iinput}\left(i-m,j-n\right),$$

In Equation (7), X can be either MRI or PET, and Equation (7) effectively captures the process of obtaining the feature maps from both images using the same convolutional operation with Kernel(m,n), which is kernel-centered at the position (m,n), Trans represents the output of transposition process, while i and j represent the row and column indices of the output feature map, respectively. The variable k is the half-size of the kernel (kernel radius), indicating the distance from the center pixel which must be considered in the summation, and Input(i−m, j−n) refers to the pixel value of the input image at the relative position (i−m, j−n).

After the transpose convolution operation, instance normalization without learnable parameters is applied across the height and width dimensions to the feature maps corresponding to MRI and PET modalities generated at the higher resolution. Instance normalization without learnable parameters is a variant of instance normalization, in which the scaling and shifting factors are not learned but are instead fixed and applied in a predetermined manner [40,41]. In this study, instance normalization helps normalize the activations of individual instances independently, without introducing any learnable parameters. Normalizing each modality ensures that the fusion process is not biased towards the modality due to differences in intensity, contrast, or scale. Also, since this instance of normalization does not require the learning of scaling and shifting parameters, it can lead to a reduction in model complexity and improve the performance of image fusion [42].

The instance normalization is represented as follows:

Step 1: The mean and variance of the feature map Trans (*i*, *j*) across the height (*H*) and width (*W*) dimensions for each instance (t) are calculated separately. Let us denote them as *μ*_*ti* and *σ*_*ti*, respectively:

(8) $$\mu_{ti}=\frac{1}{HW}\sum_{l=1}^{W}\sum_{m=1}^{H}Trans(i+m,j+n)a\;=\;1,$$

(9) $$\sigma_{ti}^{2}=\frac{1}{HW}\sum_{l=1}^{W}\sum_{m=1}^{H}{\left(Trans(i+m,j+n)-\mu_{ti}\right)}^{2}a\;=\;1,$$

Step 2: The feature map Trans(i, j) is normalized using the computed mean and variance in Equations (8) and (9) as follows:

(10) $$y_{ijk}=\frac{\left(Trans(i+m,j+n-\mu_{ti}\right)}{\sqrt{\rho_{ti}^{2}+\in }}a\;=\;1,$$

where $y_{ijk}$ is the normalized output at position (i, j) and channel (k), Trans(i+m, j+n) represents the pixel value of the feature map at the relative position $(i+m,\;j+n),\;\mu_{ti}$ and $\rho_{ti}^{2}$ are the mean and variance computed in step 1, and ε is a small constant added to the denominator to avoid division by zero. Note: the normalization is applied separately to both the MRI and PET feature maps to ensure that each feature map is normalized across its channel dimensions.

### 2.4. Basic Image Fusion Strategy Using the MS

After normalizing the feature maps separately for MRI and PET using instance normalization, MS is applied to combine the normalized feature maps. This strategy aims to leverage complementary information from both MRI and PET modalities. The basic idea is to take the maximum value between the corresponding elements of the normalized MRI and PET feature maps, pixel-wise, to create a fused feature map [43].

For each pixel position (i, j) and channel (k), the maximum value between the normalized MRI and PET feature maps is:

(11) $${Fused\;feature\;map}_{ijk}=\max({ymri}_{ijk},{ypet}_{ijk}),$$

This Maximum Strategy ensures that the fused feature map retains the strongest features from both the MRI and PET modalities, leveraging the strengths of each image while minimizing the impact of less relevant information. The fused feature map is further fed into a Vision Transformer for the classification of AD stages.

## 3. Experiment and Result Analysis

**Figure 6:**
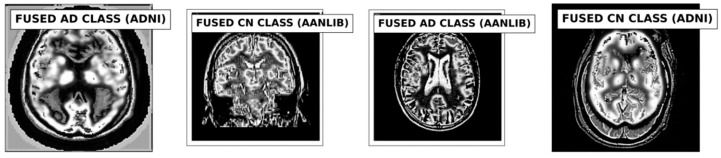
Sample of Fused Images.

To verify the effectiveness and advancement of the proposed fusion method in different scenarios, we conducted a rigorous set of evaluations and experiments that leveraged its intrinsic adaptability and generalization capabilities. Notably, our approach capitalizes on the unique ability to fuse information from single data instances, rendering it suitable for scenarios where large training datasets might be unavailable or impractical to create. By focusing on single data instances without requiring prior training, we ensured that the method’s performance was consistently assessed across three different databases. We adopted the same network size in [36] for the fusion process. To effectively assess its performance, we adopted a holistic approach based on quantitative metrics assessments. Quantitative analysis involves calculating relevant performance metrics, such as PSNR, SSIM, FSIM, E, and EBS, among others [44,45]. These metrics allowed us to quantitatively measure the fidelity, preservation of salient features, and alignment with ground truth information. The PSNR values indicate the quality of the fused images in terms of noise and distortion, where higher values suggest better quality. The SSIM values indicate the structural similarity between the original and fused images, with values closer to 1 indicating better similarity. The metrics E, FSIM, and EBS provide insights into various aspects of image quality, such as edge preservation and structural information. These metrics assess the performance of the fusion strategy in different dimensions. At the same time, the proposed algorithm will be compared with the results of the other two typical fusion methods on the dataset from the selected databases. Sample fused images are displayed in Figure 6.

**Figure 7:**
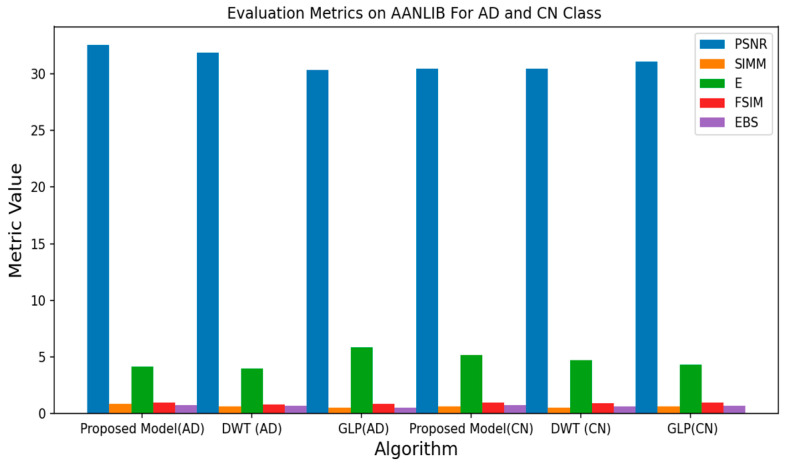
Average Metrics Value of AANLIB.

**Figure 8:**
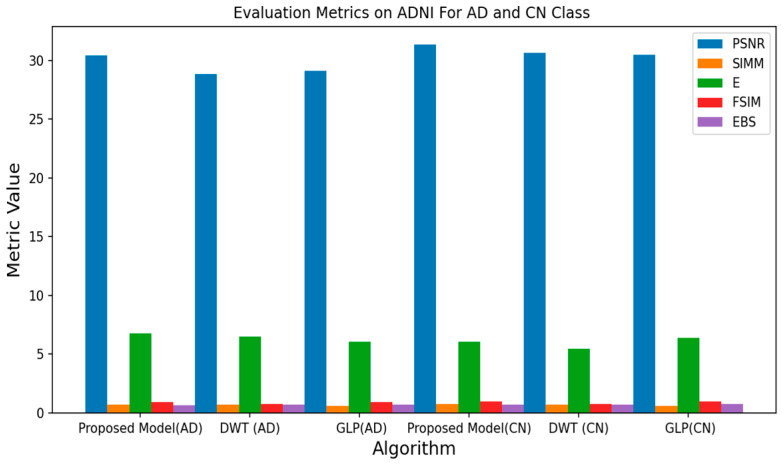
Average Metrics Value of ADNI.

**Figure 9:**
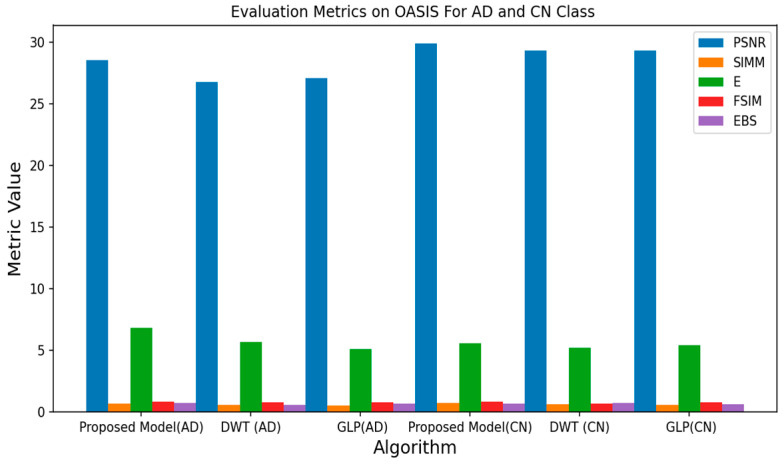
Average Metrics Value of OASIS.

All experiments were conducted using the Python programming language and executed on a GPU-accelerated system. Table 1, Table 2 and Table 3 depict the quantitative evaluation values of the fusion images corresponding to twelve typical source images from AD stages in the selected databases. The overall evaluation metrics evaluate the SSIM, FSIM, E, EBS, and PSNR of the proposed fusion model, and multiscale transform methods are depicted in Figure 7, Figure 8 and Figure 9. To assess the generalization capability and performance of the fused data on individual modalities, MViT is trained and validated using the fused data and then tested with unseen fused data from ADNI and AANLIB. The MViT model is trained on the fused data from ADNI and AANLIB separately, i.e., 150 fused data from ADNI and 100 fused data from AANLIB, as shown in Table 4. This study leveraged the hyperparameters in [29] and finetuned them for the proposed model AD stage classification. Hyperparameters used have a learning rate of 0.0002 and a weight decay of 0.01. The stochastic gradient descent optimizer performed better than the adaptive optimizer with weight decay in our study.

**Figure 10:**
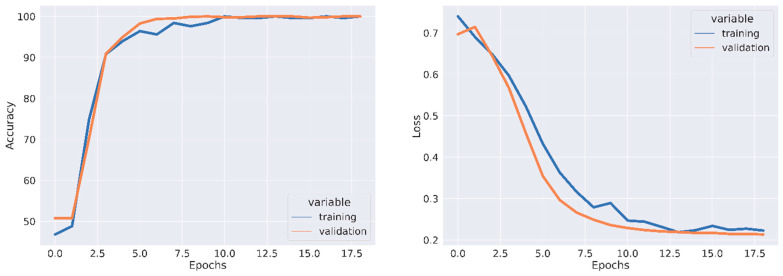
Training Accuracy, Validation Accuracy, Training Loss, and Validation Loss using Fused Data from AANLIB.

**Figure 11:**
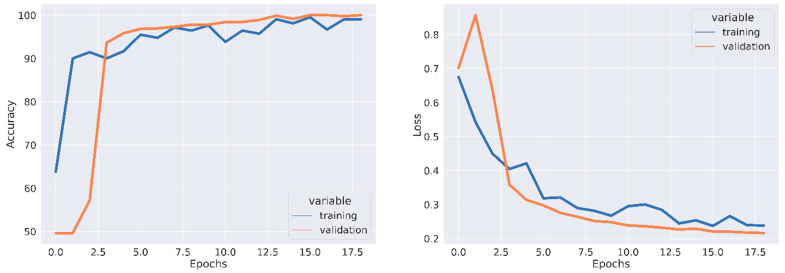
Training Accuracy, Validation Accuracy, Training Loss, and Validation Loss using Fused Data from ADNI.

**Figure 12:**
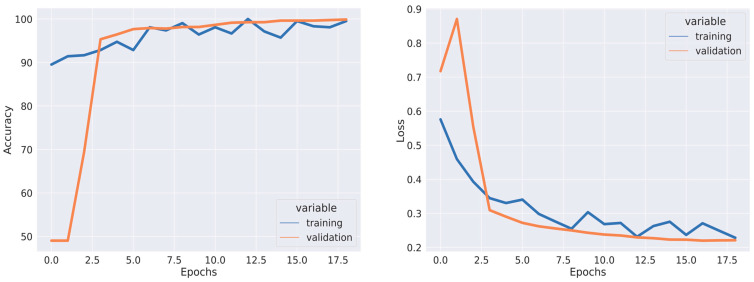
Training Accuracy, Validation Accuracy, Training Loss, and Validation Loss using Fused Data from OASIS.

**Figure 13:**
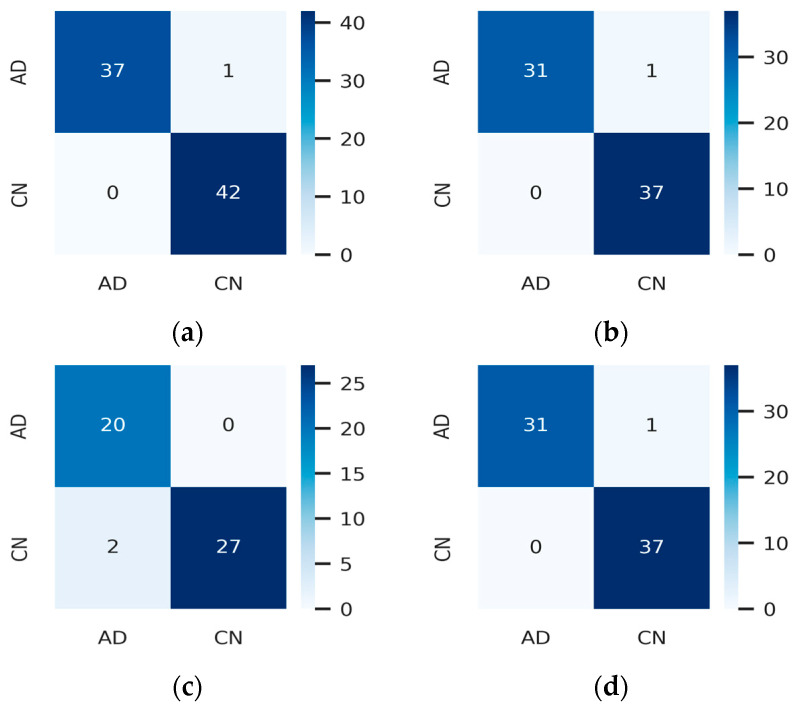

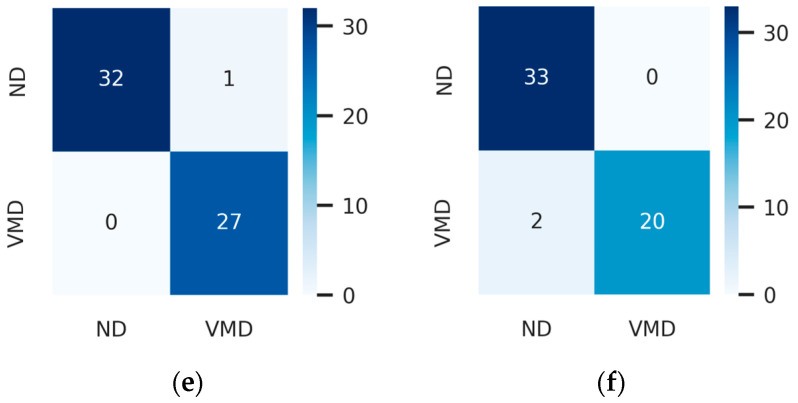
Confusion Matrix for (**a**) MRI Test Data from AANLIB, (**b**) PET Test Data from AANLIB, (**c**) MRI Test Data from ADNI, (**d**) PET Test Data from ADNI, (**e**) MRI Test Data from OASIS, (**f**) PET Test Data from OASIS.

**Figure 14:**
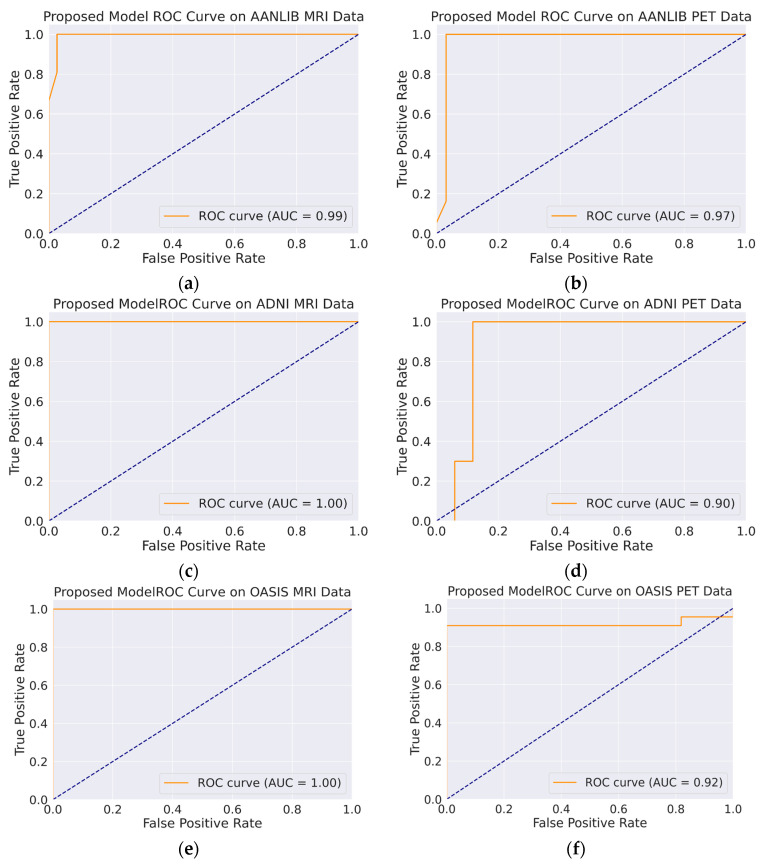
ROC Curve for Proposed Model for Classification of AD vs. CN: (**a**) AANLIB MRI Test Data, (**b**) AANLIB PET Test Data, (**c**) ADNI MRI Test Data, (**d**) ADNI PET Test Data, (**e**) OASIS MRI Test Data, (**f**) OASIS PET Test Data.

A set of image transformations was applied to the fused images to augment the dataset to minimize overfitting. The transformations were implemented in the Pytorch transforms module to provide a range of image manipulation. The training and validation accuracy/loss is depicted in Figure 10, Figure 11 and Figure 12. The Confusion matrix is used to provide insight into the model’s classification accuracy and potential misclassifications across different classes. Figure 13 depicts the confusion matrix of the proposed model. Simultaneously, the ROC curve is depicted in Figure 14, which illustrates the trade-off between a true positive rate and a false positive rate, aiding in the determination of a suitable decision threshold.

## 4. Discussion

From Table 1, it can be observed that the proposed fusion strategy generally performs better compared with DWT and LPG in terms of most metrics across both datasets. This indicates that the proposed fusion method leveraging the VGG19 model tends to preserve more image details, maintain better structural similarity, and produce higher-quality fused images. Furthermore, the results demonstrate that the proposed fusion strategy exhibits effectiveness and advancement across AD and CN without the need for explicit model training. Likewise, the results in Table 2 also show consistency in the performance trend across different stages of AD from ADNI. This suggests the robustness and general applicability of the proposed fusion method across diverse datasets, showcasing its effectiveness for various image fusion scenarios.

Figure 7, Figure 8 and Figure 9 show the proposed model achieves a PSNR of 32.55, a SIMM of 0.83, an E of 4.13, an FSIM of 0.96, and an EBS of 0.75 on the AANLIB dataset. Comparing these metrics with the other fusion models, the proposed model outperforms DWT and GLP across most metrics, indicating superior image quality and feature preservation. On the ADNI dataset, the proposed model achieves a PSNR of 30.39, a SIMM of 0.70, an E of 6.78, an FSIM of 0.93, and an EBS of 0.67. Again, the proposed model generally performs better than the other methods, showcasing its potential for accurately fusing MRI and PET data. The proposed model scores a PSNR of 28.58, a SIMM of 0.67, an E of 6.83, an FSIM of 0.85, and an EBS of 0.71 on the OASIS dataset. While still performing favorably, the differences in metrics between the models are less pronounced on this dataset. Across all three datasets, the proposed model consistently outperforms both DWT and GLP. This implies that the proposed fusion method captures more meaningful information from both MRI and PET modalities, resulting in images that are better suited for subsequent analysis or diagnosis. While DWT and GLP might have their advantages, the results indicate that the proposed model is more effective for the specific task of fusing MRI and PET data for AD vs. CN classification.

The clinical implication of these results lies in the ability of the image fusion model to aid in the classification of individuals with AD vs. those who are CN. Generally, higher performance metrics (like higher PSNR, SIMM, and FSIM values and lower E and EBS values) indicate better image fusion quality. This better fusion quality can potentially enhance the ability to detect patterns and features that are crucial for accurate classification. Figure 12 shows that the performance of the fused image in the classification of AD vs. CN using MRI test data from AANLIB provides a precision of 100% (AD) and 98% (CN), with a recall of 97% (AD), and 100% (CN), and an F1-score of 99% (AD), with 99% (CN) indicating that the model performs very well on the AANLIB MRI test dataset. It has high precision, recall, and F1-score values for both classes, which suggests that the model is effective in correctly classifying instances from both classes. The model performance using the AANLIB PET test dataset is like the AANLIB MRI dataset. The model’s performance on the ADNI MRITEST dataset is good but not as high as the AANLIB datasets. The performance of the fused image in the classification of AD vs. CN using MRI test data from ADNI provides precision values of 91% (AD) and 100% (CN), recalls of 100% (AD) and 93% (CN), and F1-scores of 95% (AD) and 96% (CN). The model’s performance on the ADNI PET test dataset is like that of the ADNI MRI test dataset. It achieves high precision and recall for both classes, but the recall for class AD is comparatively low (88%), affecting the overall F1-score for that class.

However, there are some variations in performance across datasets. The model performs slightly better on the AANLIB datasets compared with the ADNI datasets. Additionally, the performance metrics for class AD are relatively low compared with those for class CN in some cases, suggesting that the model might struggle more with the AD class. Figure 14 shows that the AUC values, which measure the model’s ability to discriminate between positive and negative classes, are generally high. The AANLIB MRI and AANLIB PET datasets have the highest AUC values, suggesting excellent discriminative power. The ADNI MRITEST dataset has perfect AUC (100%) but slightly lower accuracy compared with the AANLIB datasets. Likewise, the OASIS MRI Test data have perfect AUC (100%). The ADNI PET dataset has a relatively low AUC (90%) compared with the other datasets, which might indicate that the model has slightly more difficulty distinguishing between classes. The high recall values indicate that the model is effective at identifying most samples of the positive class, which is crucial in medical diagnosis in order to avoid false negatives. The high AUC values imply that the model can effectively distinguish between different classes, which is vital for reliable diagnostic decisions. However, while the model’s performance is promising, in clinical decisions, the model should be used as a supportive tool for medical professionals to aid in diagnosis and decision making. A summary of the proposed model performance is depicted in Table 5.

The results achieved in this article are compared and validated with the recent research conducted on AD detection using multimodal neuroimaging in Table 5.

Table 6 presents a comparison between the proposed method and several existing methods for binary classification tasks in AD vs. CN classification using the ADNI database. The proposed method demonstrates competitive performance compared with existing methods. While some methods achieve higher accuracy, the proposed method maintains a good balance between specificity and sensitivity, which is crucial in medical diagnosis scenarios. Additionally, the proposed method’s performance on the AANLIB dataset suggests its potential for generalizability across different datasets.

## 5. Conclusions

In this research study, we proposed a novel fusion method for multimodal neuroimaging data consisting of MRI and PET images to enhance the accuracy of Alzheimer’s Disease classification. The method leveraged a Maximum Fusion strategy and an optimized convolution network, effectively combining the complementary information from both modalities. The fusion approach demonstrated its effectiveness across multiple datasets and AD stages without the need for explicit model training. Our comprehensive experiments and result analysis showcased the superior performance of the proposed fusion method compared with traditional fusion methods such as Discrete Wavelet Transform and Laplacian Pyramid Gaussian in terms of various quantitative metrics. The fused images were used to train the Mobile Vision Transformer for the classification of Alzheimer’s Disease vs. Cognitive Normal classification, and the proposed model’s classification accuracy, precision, recall, F1-score, and AUC values demonstrated its effectiveness.

Comparisons with existing methods highlighted the competitive nature of our proposed approach, maintaining a good balance between the specificity and sensitivity crucial for medical diagnosis scenarios. The fusion model’s clinical implication lies in its potential to aid medical professionals in the accurate classification of AD vs. CN patients. One major limitation is the absence of real clinical data for validation, which could impact the model’s performance when applied to real-world scenarios. Additionally, the proposed model’s performance might be influenced by variations in data acquisition protocols and scanner characteristics in clinical settings. Future research could focus on validating the method using diverse clinical datasets and addressing the limitations observed in this study.

## Figures and Tables

**Table 1:** Quantitative Evaluation Values of Fused Images from AANLIB Database.

Image	PSNR			SSIM			E			FSIM			EBS		
	DWT	LPG	Proposed	DWT	LPG	Proposed	DWT	LPG	Proposed	DWT	LPG	Proposed	DWT	LPG	Proposed
AD															
1	31.95	30.29	33.30	0.66	0.50	0.74	3.98	5.89	4.11	0.83	0.90	0.99	0.73	0.50	0.86
2	31.80	30.26	31.40	0.66	0.50	0.80	4.02	5.84	4.15	0.83	0.90	0.99	0.70	0.47	0.76
3	31.80	30.28	32.90	0.54	0.50	0.90	3.98	5.84	4,12	0.83	0.90	0.90	0.68	0.51	0.79
4	31.90	30.44	32.90	0.54	0.50	0.90	3.92	5.84	4.14	0.83	0.90	0.99	0.67	0.48	0.69
5	31.80	30.28	32.90	0.55	0.50	0.80	4.02	5.89	4.11	0.83	0.80	0.88	0.70	0.51	0.73
6	31.80	30.44	32.50	0.55	0.55	0.83	3.98	5.89	4.14	0.83	0.92	0.99	0.67	0.48	0.72
7	31.80	30.28	32.00	0.58	0.50	0.83	3.98	5.84	4.11	0.83	0.80	0.89	0.68	0.48	0.75
8	31.90	30.44	32.00	0.58	0.50	0.83	3.92	5.84	4.14	0.83	0.90	0.99	0.67	0.51	0.69
9	31.80	30.26	32.90	0.55	0.50	0.87	3.92	5.89	4.14	0.83	0.80	0.99	0.69	0.50	0.79
10	31.95	30.44	32.00	0.70	0.55	0.87	4.02	5.89	4.11	0.82	0.90	0.99	0.73	0.50	0.80
11	31.95	30.39	32.90	0.72	0.54	0.86	4.02	5.84	4.14	0.83	0.90	0.98	0.71	0.50	0.75
12	31.95	30.39	32.90	0.70	0.50	0.83	3.98	5.84	4.14	0.82	0.90	0.99	0.72	0.51	0.75
CN															
1	30.95	31.65	30.99	0.52	0.60	0.62	4.74	4.29	5.45	0.93	0.98	1.00	0.63	0.66	0.70
2	30.88	31.65	3104	0.52	0.60	0.62	4.74	4.29	5.05	0.93	0.97	0.99	0.64	0.66	0.72
3	30.84	30.95	31.80	0.54	0.66	0.68	4.74	4.40	5.00	0.93	0.97	0.99	0.63	0.66	0.73
4	30.95	31.00	31.80	0.52	0.66	0.68	4.74	4.29	5.08	0.98	0.98	0.99	0.63	0.66	0.73
5	30.95	30.95	31.80	0.55	0.67	0.68	4.74	4.40	5.18	0.98	0.98	0.99	0.63	0.66	0.70
6	31.08	30.95	31.67	0.52	0.68	0.67	4.74	4.29	5.18	0.93	0.98	0.99	0.64	0.66	0.73
7	31.08	31.00	31.67	0.52	0.66	0.67	4.74	4.44	5.24	0.94	0.98	0.99	0.64	0.66	0.73
8	30.85	31.00	31.08	0.52	0.65	0.67	4.74	4.29	5.15	0.94	0.97	0.99	0.63	0.67	0.70
9	30.90	31.05	31.08	0.52	0.66	0.62	4.74	4.29	5.33	0.93	0.98	1.00	0.63	0.67	0.73
10	30.95	31.05	31.65	0.52	0.65	0.65	4.75	4.29	5.14	0.94	0.98	1.00	0.63	0.66	0.73
11	30.95	31.05	31.75	0.52	0.65	0.65	4.74	4.24	5.00	0.94	0.98	0.99	0.63	0.66	0.70
12	30.95	31.05	31.04	0.52	0.60	0.60	4.74	4.30	5.25	0.94	0.97	0.99	0.63	0.66	0.70

PSNR = Peak Signal-To-Noise Ratio (PSNR), SSIM = Structural Similarity Index Measure (SSIM), E = Entropy, FSIM = Feature Similarity Indexing Method, EBS = Edge-Based Similarity, DWT = Discrete Wave Transform, LPG = Laplacian Gaussian Pyramid, AD = Alzheimer’s Disease, and CN = Cognitive Normal.

**Table 2:** Quantitative Evaluation Values of Fused Images from the ADNI Database.

Image	PSNR			SSIM			E			FSIM			EBS		
	DWT	LPG	Proposed	DWT	LPG	Proposed	DWT	LPG	Proposed	DWT	LPG	Proposed	DWT	LPG	Proposed
AD															
1	28.60	29.98	29.00	0.68	0.53	0.70	6.50	5.90	6.90	0.80	0.93	0.85	0.71	0.71	0.70
2	28.32	28.83	29.01	0.68	0.60	0.70	6.50	5.90	6.90	0.80	0.97	0.99	0.71	0.73	0.69
3	28.32	28.83	30.00	0.68	0.60	0.70	6.50	6.04	6.70	0.77	0.95	0.90	0.73	0.71	0.69
4	28.82	29.08	30.25	0.68	0.60	0.70	6.50	6.04	6.60	0.77	0.93	0.99	0.72	0.73	0.65
5	28.82	29.09	30.56	0.68	0.64	0.70	6.50	6.05	6.90	0.78	0.93	0.90	0.71	0.75	0.65
6	28.82	29.14	30.56	0.69	0.60	0.70	6.50	6.05	6.90	0.78	0.98	0.90	0.71	0.71	0.69
7	28.82	29.07	30.00	0.69	0.60	0.70	6.50	6.20	6.80	0.78	0.90	0.95	0.71	0.60	0.69
8	28.82	29.06	30.05	0.69	0.62	0.70	6.50	6.20	6.75	0.79	0.90	0.96	0.71	0.71	0.65
9	29.10	29.08	30.98	0.69	0.60	0.70	6.50	6.20	6.75	0.79	0.90	0.95	0.71	0.74	0.69
10	29.10	29.14	30.98	0.71	0.60	0.71	6.50	6.20	6.75	0.78	0.90	0.95	0.71	0.60	0.65
11	29.10	29.08	30.98	0.71	0.60	0.71	6.50	6.20	6.75	0.79	0.90	0.95	0.71	0.73	0.69
12	29.10	29.07	30.98	0.71	0.60	0.71	6.50	6.20	6.75	0.79	0.95	0.90	0.71	0.74	0.69
CN															
1	31.05	30.50	31.25	0.75	0.65	0.89	5.30	6.20	6.40	0.83	0.97	0.99	0.72	0.76	0.70
2	29.24	29.53	31.09	0.72	0.60	0.87	6.30	6.90	6.36	0.80	0.96	0.97	0.70	0.73	0.69
3	31.05	30.79	31.90	0.74	0.65	0.83	4.86	7.00	5.00	0.69	0.96	0.99	0.70	0.76	0.77
4	31.22	31.15	31.55	0.78	0.62	0.79	5.08	6.30	5.00	0.70	0.96	0.99	0.76	0.76	0.76
5	29.94	29.43	32.05	0.67	0.64	0.76	6.33	6.30	6.50	0.81	0.96	0.99	0.78	0.76	0.79
6	30.44	30.35	31.05	0.70	0.60	0.84	5.35	6.32	5.53	0.78	0.98	0.97	0.70	0.76	0.69
7	30.73	30.61	30.92	0.70	0.60	0.73	5.80	6.32	6.94	0.81	0.98	0.99	0.70	0.76	0.78
8	31.06	30.87	30.94	0.72	0.61	0.76	5.26	6.41	5.60	0.74	0.98	0.96	0.75	0.76	0.74
9	30.55	30.61	30.57	0.70	0.60	0.78	5.46	6.20	6.66	0.75	0.98	0.98	0.75	0.76	0.68
10	30.49	30.61	31.73	0.70	0.60	0.71	5.33	6.30	5.53	0.80	0.96	0.99	0.70	0.76	0.69
11	30.50	30.61	31.05	0.70	0.65	0.77	5.25	6.30	6.06	0.75	0.96	0.98	0.70	0.76	0.79
12	31.60	30.64	31.90	0.70	0.66	0.81	5.28	6.30	6.90	0.89	0.96	0.98	0.72	0.78	0.73
EMCI															
1	30.95	30.45	32.05	0.74	0.63	0.88	5.45	6.15	6.60	0.82	0.96	0.98	0.74	0.77	0.71
2	30.75	30.60	31.95	0.73	0.62	0.86	5.40	6.10	6.55	0.81	0.97	0.98	0.73	0.76	0.70
3	31.15	30.75	32.15	0.75	0.64	0.89	5.55	6.20	6.70	0.83	0.97	0.99	0.75	0.78	0.72
4	30.85	30.65	31.75	0.729	0.61	0.85	5.50	6.05	6.50	0.80	0.96	0.98	0.72	0.75	0.69
5	31.05	30.50	32.00	0.74	0.63	0.87	5.60	6.25	6.65	0.82	0.97	0.99	0.74	0.77	0.71
6	30.95	30.70	31.90	0.73	0.62	0.88	5.58	6.18	6.63	0.81	0.97	0.98	0.73	0.76	0.70
7	31.10	30.75	32.10	0.75	0.64	0.89	5.63	6.23	6.68	0.83	0.97	0.99	0.75	0.78	0.72
8	30.90	30.60	31.80	0.72	0.61	0.86	5.55	6.08	6.53	0.80	0.96	0.98	0.72	0.75	0.69
9	31.00	30.55	31.95	0.74	0.63	0.88	5.58	6.15	6.60	0.82	0.97	0.99	0.74	0.77	0.71
10	31.05	30.60	32.05	0.74	0.63	0.89	5.60	6.20	6.65	0.83	0.97	0.99	0.74	0.77	0.72
11	30.85	30.65	31.75	0.72	0.61	0.86	5.53	6.10	6.55	0.80	0.96	0.98	0.72	0.75	0.70
12	31.20	30.70	32.20	0.75	0.64	0.89	5.65	6.25	6.70	0.83	0.97	0.99	0.75	0.78	0.72

PSNR = Peak Signal-To-Noise Ratio (PSNR), SSIM = Structural Similarity Index Measure (SSIM), E = Entropy, FSIM = Feature Similarity Indexing Method, EBS = Edge-Based Similarity, DWT = Discrete Wave Transform, LPG = Laplacian Gaussian Pyramid, AD = Alzheimer’s Disease, CN = Cognitive Normal, and EMCI = Early Mild Cognitive Normal.

**Table 3:** Quantitative Evaluation Values of Fused Images from the OASIS Database.

Image	PSNR			SSIM			E			FSIM			EBS		
	DWT	LPG	Proposed	DWT	LPG	Proposed	DWT	LPG	Proposed	DWT	LPG	Proposed	DWT	LPG	Proposed
VMD															
1	26.60	27.98	26.90	0.58	0.43	0.57	6.00	4.90	6.10	0.70	0.90	0.84	0.59	0.71	0.75
2	26.32	26.83	26.50	0.58	0.50	0.62	5.50	5.00	5.90	0.80	0.87	0.84	0.60	0.73	0.70
3	26.32	26.83	26.0	0.58	0.50	0.57	5.50	5.04	6.72	0.87	0.80	0.87	0.60	0.71	0.70
4	26.80	27.08	29.80	0.58	0.50	0.60	5.50	5.04	6.03	0.77	0.80	0.88	0.52	0.70	0.75
5	26.82	27.09	29.80	0.58	0.54	0.80	5.50	5.05	6.03	0.88	0.90	0.87	0.60	0.75	0.65
6	26.80	27.14	29.70	0.57	0.50	0.60	5.50	5.05	7.90	0.70	0.80	0.75	0.60	0.71	0.79
7	26.82	27.07	28.90	0.59	0.50	0.60	5.50	5.20	6.71	0.88	0.70	0.93	0.60	0.52	0.70
8	26.82	27.06	28.85	0.67	0.52	0.71	5.50	5.20	7.72	0.79	0.80	0.87	0.60	0.71	0.69
9	27.10	27.06	29.15	0.69	0.50	0.80	6.00	5.20	6.71	0.89	0.70	0.87	0.60	0.71	0.76
10	27.20	27.10	29.15	0.61	0.50	0.71	5.50	5.20	7.75	0.78	0.80	0.86	0.70	0.54	0.69
11	27.10	27.00	29.50	0.61	0.50	0.78	6.00	5.20	6.76	0.89	0.60	0.82	0.60	0.73	0.69
12	27.10	27.01	28.80	0.61	0.50	0.70	6.00	5.20	7.71	0.70	0.65	0.82	0.70	0.71	0.68
ND															
1	30.05	29.50	31.06	0.65	0.60	0.70	5.00	5.20	5.00	0.80	0.80	0.86	0.70	0.66	0.69
2	28.24	28.53	30.20	0.62	0.58	0.74	6.00	5.90	5.60	0.70	0.80	0.88	0.71	0.63	0.69
3	30.05	29.70	29.90	0.64	0.60	0.79	4.56	6.00	5.40	0.60	0.80	0.85	0.71	0.66	0.70
4	30.22	29.15	29.85	0.70	0.60	0.75	5.00	5.30	5.40	0.60	0.86	0.87	0.75	0.66	0.70
5	28.90	28.40	30.00	0.57	0.60	0.64	6.30	5.30	6.40	0.71	0.70	0.88	0.76	0.66	0.70
6	28.00	29.30	30.00	0.60	0.58	0.78	5.30	5.32	5.60	0.70	0.80	0.86	0.71	0.66	0.70
7	28.70	29.60	29.90	0.69	0.57	0.65	5.20	5.32	5.70	0.71	0.90	0.79	0.71	0.66	0.70
8	30.00	29.80	30.00	0.62	0.60	0.69	5.20	5.41	5.60	0.70	0.78	0.89	0.73	0.66	0.69
9	29.00	29.60	29.50	0.69	0.50	0.66	5.40	5.20	5.70	0.70	0.80	0.86	0.74	0.66	0.69
10	29.40	29.60	29.63	0.65	0.58	0.60	4.33	5.30	5.73	0.70	0.86	0.87	0.71	0.66	0.69
11	29.40	29.60	29.00	0.65	0.60	0.77	5.20	5.30	5.66	0.70	0.80	0.86	0.74	0.66	0.69
12	30.40	29.54	29.80	0.65	0.60	0.78	5.20	5.30	5.70	0.80	0.86	0.79	0.70	0.68	0.75
MD															
1	30.00	29.80	30.25	0.60	0.50	0.63	5.20	5.80	5.51	0.70	0.76	0.87	0.60	0.40	0.59
2	30.00	28.80	30.90	0.60	0.50	0.59	5.20	5.80	6.02	0.85	0.86	0.92	0.60	0.40	0.67
3	30.01	29.80	29.50	0.70	0.50	0.56	5.78	5.80	5.05	0.85	0.90	0.75	0.52	0.43	0.70
4	29.50	29.89	30.50	0.61	0.52	0.50	5.00	5.80	5.21	0.70	0.90	0.81	0.50	0.50	0.70
5	29.50	29.00	30.60	0.61	0.52	0.69	5.40	5.05	5.06	0.70	0.85	0.81	0.50	0.50	0.69
6	29.50	31.70	31.20	0.60	0.50	0.68	5.20	5.85	6.02	0.80	0.75	0.81	0.50	0.50	0.68
7	30.50	28.10	29.90	0.65	0.50	0.68	5.20	6.05	5.24	0.70	0.70	0.88	0.50	0.50	0.66
8	30.50	30.70	29.92	0.79	0.50	0.69	6.20	5.06	5.03	0.70	0.85	0.81	0.50	0.50	0.65
9	30.05	31.70	30.45	0.50	0.40	0.61	4.30	5.07	4.06	0.70	0.90	0.92	0.50	0.50	0.66
10	30.50	29.00	28.35	0.62	0.50	0.70	6.30	5.89	5.99	0.85	0.85	0.86	0.50	0.50	0.66
11	30.50	30.00	29.35	0.63	0.50	0.69	5.30	5.56	5.99	0.71	0.90	0.81	0.50	0.50	0.65
12	30.50	30.00	30.25	0.70	0.55	0.69	3.30	5.86	5.07	0.90	0.88	0.75	0.50	0.50	0.65

PSNR = Peak Signal-To-Noise Ratio (PSNR), SSIM = Structural Similarity Index Measure (SSIM), E = Entropy, FSIM = Feature Similarity Indexing Method, EBS = Edge-Based Similarity, DWT = Discrete Wave Transform, LPG = Laplacian Gaussian Pyramid, VMD = Very Mild Dementia, ND = Non Dementia, MD = Mild Dementia.

**Table 4:** Details of Source Images, Fused Images, and Augmented Images.

Image	No of Images							
	ADNI			AANLIB		OASIS		
	AD	CN	EMCI	AD	CN	CN	VMD	MD
Fused	50	50	50	50	50	50	50	50
Augmented	1000	1000	1000	1000	1000	1000	1000	1000

**Table 5:** Summary of Proposed Model Performance.

	AANLIB MRI Test Data	AANLIB PET Test Data	ADNI MRI Test Data	ADNI PET Test Data	OASIS MRI Test Data	OASIS PET Test Data
Accuracy (%)	99.00	99.00	95.91	95.74	98.30	96.30
Precision (%)	99.00	98.50	95.50	97.00	98.00	97.00
Recall (%)	98.50	98.50	96.50	94.00	98.50	95.50
F1-Score (%)	99.99	98.50	95.50	95.50	98.00	96.00
Sensitivity (%)	96.88	100	100	88.24	100	94.29
Specificity (%)	100	97.37	93.10	100	96.43	100

**Table 6:** Comparison of Proposed Method with Existing Methods.

Reference	Database	Method	Binary Classification	Accuracy (%)	Specificity (%)	Sensitivity (%)
[17]	ADNI	Feature level fusion + Ensemble Classifier	AD vs. CN	99.00	-	-
[18]	ADNI	Hypergraph-based Regularization	AD vs. CN	92.51	90.44	94.08
[19]	ADNI	3D CNN with Sparse Autoencoder	AD vs. CN	93.21	95.42	91.43
[22]	ADNI	Inception–Resnet with DWT	AD vs. CN	97.00	97.00	97.00
[23]	ADNI	DWT + Pretrained ViT	AD vs. EMCI	93.75	-	-
Proposed	ADNI	Optimized Transposition + Mobile ViT	AD vs. CN	96.00	97.00	94.12
	AANLIB			99.00	99.00	98.44

## Data Availability

Not applicable.
